# Nesprin-1 has key roles in the process of mesenchymal stem cell differentiation into cardiomyocyte-like cells *in vivo* and *in vitro*

**DOI:** 10.3892/mmr.2014.2754

**Published:** 2014-10-23

**Authors:** WENGANG YANG, HUI ZHENG, YONGYI WANG, FENG LIAN, ZHENGLEI HU, SONG XUE

**Affiliations:** Department of Cardiovascular Surgery, Ren Ji Hospital, School of Medicine, Shanghai Jiao Tong University, Shanghai 200127, P.R. China

**Keywords:** mesenchymal stem cells, cardiomyocyte-like cells, nesprin-1 protein, differentiation, myocardial ischemia

## Abstract

The aim of the present study was to investigate the expression of nesprin-1 protein in MSCs and its effects on the differentiation of rat bone-marrow mesenchymal stem cells (MSCs). Surface-associated antigens of MSCs were detected by flow cytometry. MSC differentiation was induced by treatment with 10 μmol/l 5-azacytidine. Sprague-Dawley rats were anesthetized prior to thoracotomy and subsequent ligation of the left anterior descending coronary artery to establish a model of myocardial infarction. Two weeks following myocardial infarction, DAPI-marked MSCs were injected into the infarcted region in the experimental group, while DMEM was injected into the infarcted region of the control group. Characteristics of the putative cardiac-myogenic cells were evaluated using immunohistochemical and immunofluorescent analysis. The messenger RNA expression levels of cardiac-myogenic specific genes; desmin, α-actinin and cardiac troponin I (cTnI) were detected by reverse transcription quantitative polymerase chain reaction. The expression of nesprin-1 protein in MSCs was identified by immunofluorescence and western blot analysis, prior to and following MSC differentiation. Following differentiation, the MSCs appeared spindle-shaped with irregular processes and were positive for CD90 and CD29, but negative for CD45. Cardiomyocyte-like cells were positive for desmin, α-sarcomeric actin and cTnI. Nesprin protein was detected in the nuclear membrane via immunofluorescence, and following MSC differentiation into cardiomyocyte-like cells, the expression of nesprin protein was significantly higher (^*^P=0.03<0.05). The results of the present study indicated that MSCs may be differentiated *in vitro* and *in vivo* into cells with characteristics commonly attributed to cardiomyocytes. Cardiomyocyte-like cells cultured from bone marrow sources may be potentially useful for repairing the injured myocardium. The results also suggested that, consistent with the results of previous studies, the expression of nesprin-1 protein was higher during the differentiation process of MSCs and may have an important role in mediating MSC differentiation. Elucidation of the role of nesprin-1 in MSC differentiation may aid in the development of novel therapies for the treatment of myocardial ischemia and nesprin-1 genetic deficiencies.

## Introduction

Nesprin-1 protein is a nuclear membrane protein expressed in numerous tissues. In particular, the expression of nesprin-1 expression levels are higher in skeletal muscles and cardiac and vascular smooth muscles than those in other tissues (1). It has been reported that nesprin-1 not only has a key role in cell mitosis (2), RNA copy of transport and controlling the stability of the nuclear membrane, but may also have an important role in mediating cell differentiation (3,4). Deficiencies in nesprin-1 or nesprin-2 protein may lead to muscle-associated diseases, including dilated cardiomyopathy and emery-dreifuss muscular dystrophy (5). Deficiencies may also cause cytoskeletal reorganization and disorder of dynamic balance, which results in a loss of cytoskeletal rigidity. The study of nesprin-1 may therefore aid the evaluation of potential therapeutic measures for certain genetic diseases, including syndromes characterized by a deficiency of the muscular or cardiovascular systems.

Myocardial ischemia may result in significant myocardial dysfunction or even heart failure. Though pharmacological and surgical treatments available may reduce tissue ischemia, the damaged myocardial cells cannot be replaced (6). Mesenchymal stem cells (MSCs) are bone marrow-derived cells that retain the capability to differentiate into various types of tissue cells and contribute to the regeneration of a variety of mesenchymal tissues, including bone, cartilage, muscle and adipose tissue (5–7). MSCs, transplanted into ischemic myocardial tissue, are able to secrete a variety of factors including vascular endothelial growth factor and therefore improve cardiac performance (8). The cardio-protective effects of MSCs are known to be mediated by their differentiation into vascular cells and cardiomyocyte-like cells and additionally by their ability to supply large quantities of angiogenic, anti-apoptotic and mitogenic factors (9–10). These results suggested that MSCs have therapeutic potential for the treatment of heart failure.

Recently, it was reported that MSCs were able to be differentiated into cardiomyocyte-like cells *in vivo* and *in vitro* under specific conditions (7); however, the mechanisms underlying the differentiation process has remained to be elucidated. The present study aimed to investigate the expression of nesprin-1 protein and its effects on the differentiation of rat bone-marrow MSCs *in vitro* and *in vivo*. MSC differentiation was induced by 5-azacytidine treatment *in vitro* and the expression of various structural proteins and nesprin-1 was analyzed. MSCs were subsequently transplanted into an animal model of myocardial infarction and the expression of structural genes and proteins was further analyzed *in vivo*.

## Materials and methods

### Animals

Sprague-Dawley (SD) rats, weighing 250–300 g, were obtained from the Medicine Animal Experimental Center of Shanghai Jiao Tong University School (Shanghai, China). They were in normal circadian, 26–27°C conditions and fed with normal feed. Production license: scxk(hu)2004-0001, use license number: syxk(hu)2003–2009. The experimental protocol was reviewed and approved by the University of Shanghai Institutional Animal Care and Use Committee (Shanghai, China). Procedures were performed in accordance with the guidelines for animal experimentation of Shanghai Jiaotong University, and were approved by the Shanghai Jiaotong University Ethics Committee (Shanghai, China).

### Reagents

Except where otherwise specified, all reagents were purchased from Sigma-Aldrich (St. Louis, MO, USA) and Gibco-BRL (Invitrogen Life Technologies, Carlsbad, CA, USA). Cell culture mediums, low-glucose Dulbecco’s modified Eagle’s medium (DMEM) and fetal bovine serum (FBS), were purchased from Gibco-BRL. The cardiac-specific antibodies (cTnI, α-sarcomeric actin, actinin and desmin), fluorescein isothiocyanate (FITC)-conjugated goat anti-rat antibodies (CD45), phycoerythrin (PE)-conjugated rabbit anti-rat antibodies (CD90), allophycocyanin (APC)-conjugated rabbit anti-rat antibodies (CD29) and FITC-conjugated rabbit anti-rat nesprin-1 antibodies were purchased from Abcam (Cambridge, UK). The reagents and instruments for immunohistochemistry, immunofluorescence and western blot analysis were purchased from the Gibco-BRL, Abcam, Qiagen (Santa Clara, CA, USA) and Roche Diagnostics (Mannheim, Germany).

### Cell culture

Eight-week-old SD rats (250–300 g) were used as donors. Under general anesthesia with ether, ~100 μl bone marrow was aspirated from the tibia and femur with a 20-gauge needle attached to a 10-ml syringe containing 0.5 ml DMEM and 40 U/ml heparin. Following aspiration, a cell suspension was obtained by passing the aspirate through syringe needles of decreasing sizes.

The concentration of cells in suspension was adjusted to 5×10^5^ mononuclear cells/ml DMEM, supplemented with 20% FBS, penicillin (100 U/ml) and streptomycin (100 μg/ml) at 37°C in humid air with 5% CO_2_. The cells were subsequently seeded on culture plates, without removal of red blood cells. BMSCs initially grow in colonies and do not reach confluence over the entire culture dish; therefore, the cells were first passed seven days following seeding, when half the colonies had reached 70–80% confluence. The subsequent passages were performed weekly, when the cells reached confluence. For subcultures, adherent BMSCs were harvested using 0.125% trypsin and plated at a ratio of 1:3.

For flow cytometry experiments, cells were detached using accutase, for enhanced preservation of the cell surface molecules.

Twenty-four hours following seeding of freshly isolated BMSCs, 10 μmol/l 5-azacytidine was added to the culture medium. Following incubation for a further 24 h, the BMSCs were washed and further cultured in fresh medium that was changed every 48 h. The 5-azacytidine treatment was repeated two or three times, depending on the specific experiment. In the control group, BMSCs were cultured under the same conditions except that 5-azacytidine exposure was omitted.

### Labeling of MSCs

Following passage, two batches of cells became almost confluent. Sterile DAPI solution was added to the culture medium on the day of implantation at a final concentration of 50 mg/ml for 30 min (11). The MSCs were subsequently rinsed six times in PBS to remove excess, unbound DAPI. The MSCs were harvested (~1×10^6^ cells for each implantation) and resuspended in 50 μl serum-free DMEM.

### Myocardial infarction model and stem cell transplantation

SD rats were intubated under general anesthesia using 4% chloral hydrate (4 mg/kg, administered intraperitoneally) and ventilated with room air using a small animal ventilator (Zhejiang University Apparatus, Hangzhou, China). Myocardial infarction was induced by ligation of the left anterior descending coronary artery 2–3 mm from the tip of the left auricle with a 7-0 silk suture (12). Successful coronary occlusion was verified by blanching of the myocardium distal to the coronary ligation. The sham-operation group received the same thoracotomy procedure without coronary ligation. The rats were divided randomly into three groups (8 rats per group): Sham-operation group; MSC group where rats received an MSC transplant two weeks following myocardial infarction by injection of 1×10^6^ cells in 50 μl DMEM directly into the infarct border zone; and the DMEM group, where rats received an injection of an identical volume of DMEM as the transplant subjects omitting the MSCs.

### Flow cytometric analysis

Flow cytometry was performed using a FACSAria (Beckton-Dickinson, BD Biosciences, San Jose, CA, USA) flow cytometer/cell sorter. Following accutase treatment, cells were resuspended at a density of 10^5^ cells/200 μl PBS with 2% fetal calf serum (PBS-FCS) and incubations were performed on ice. For each antibody used, 1×10^5^ cells were stained. Cells were incubated with the FITC-conjugated CD45 monoclonal antibody, PE-conjugated CD90 monoclonal antibody or APC-conjugated CD29 monoclonal antibody (at the concentrations indicated by the manufacturer) for 30 min at 4°C in the dark and subsequently washed in PBS-FCS. Following washing, cells were analyzed using the flow cytometer. A minimum of 5,000 events were analyzed for each sample. Negative controls, used to detect unspecific binding, included an irrelevant antibody or PBS-FCS alone. Data were analyzed using Summit™ 5.2 software (Cytomation, Inc., Fort Collins, CO, USA).

### Reverse transcription quantitative polymerase chain reaction (RT-qPCR)

Total RNA (0.5 μg) was isolated using the guanidinium method (13) and was reverse-transcribed in a 21-μl reaction mixture that contained 75 mmol/l KCl, 50 mmol/l Tris-HCl (pH 8.3), 3 mmol/l MgCl_2_, 0.5 mmol/l each of deoxyadenosine triphosphate (dATP), deoxycytidine triphosphate (dCTP), deoxyguanosine triphosphate (dGTP) and deoxythymidine triphosphate (dTTP), 600 ng random hexamer primers, 10 mmol/l dithiothreitol, 2 U RNAse inhibitor and 10 U Superscript RNase H (Invitrogen Life Technologies) according to the manufacturer’s instructions. 3-μl aliquots of total cDNA were amplified (Mastercycler; Eppendorf, Hamburg, Germany) in a 25-μl reaction mixture containing 50 mmol/l KCl, 10 mmol/l Tris-HCl (pH 8.3), 1.5 mmol/l MgCl_2_, 0.2 mmol/l each of dATP, dCTP, dGTP and dTTP, 25 pmol each forward and reverse primer and 1.25 U of Taq polymerase (Applied Sangon Biotech Co., Ltd, Shanghai, China). The same single-stranded cDNA product was used to analyze the expression of all genes described. To assure that amplification was in the exponential range, PCR progress was determined by amplifying identical reaction mixtures for ascending numbers of cycles. Following the cited number of PCR cycles, the amplification rate was sufficient without reaching saturation for any of the amplicons. PCR products were resolved using 2% agarose gel electrophoresis and stained with ethidium bromide. Bands imaged by a CCD camera (Biostep GmbH, Jahnsdorf, Germany) were analyzed via optical densitometry with Phoretix Grabber 3.01 and Phoretix Totallab 1.00 image processing and analying software (Biostep GmbH, Jahnsdorf, Germany). The primers (Table I) were designed by Sangon Biotech Co., Ltd (Shanghai, China) and the experiment was run three times. As a control, the 530-bp band corresponding to human β-actin transcript was amplified. α-actinin, desmin and cTnI mRNA expression levels were calculated as the ratio of the intensity of the corresponding band to the β-actin band by densitometry.

### Immunohistochemical analysis

The MSCs and the cardiomyocyte-like cells that differentiated from the MSCs adherent to chamber slides were fixed for 10 min with methanol at −20°C. Following washing three times with PBS, the cells were incubated at 4°C overnight with the primary antibodies directed against cardiac-specific proteins, including cTnI, desmin and α-sarcomeric actin. The cells were subsequently incubated with the secondary antibodies at 37°C for 30 min, prior to incubation with diaminobenzidine (DAB) reagent for 5–10 min. Finally, the cells were mounted for microscopic examination with neutral gum and cells exhibiting a brown granular DAB/ H_2_O_2_ reaction product in the cytoplasm were considered positive for the protein in question.

### Immunofluorescence microscopy

BMSCs grown on glass coverslips were fixed by 20-min incubation in 4% formaldehyde (freshly prepared from paraformaldehyde), rinsed in PBS and stored in 70% ethanol at −20°C. The fixed cells were blocked for 30 min in blocking solution (PBS supplemented with 2% goat serum, 1% BSA, 0.1% gelatin, 0.1% Triton X-100, and 0.05% Tween 10) and incubated overnight with the primary antibody (at the dilution indicated by the manufacturer) at 4°C. Following washing, the cells were incubated with the secondary antibody (FITC-conjugated anti-rat immunoglobulin G (IgG) for cTnI and desmin; α-sarcomeric actin, and FITC-conjugated anti-rat IgG for nesprin-1, respectively) for 30 min. Finally, the coverslips were washed, mounted in glycerol and examined under an epifluorescence microscope (Olympus Corp., Tokyo, Japan).

The subsets of animals were killed three weeks following MSC transplantation (n=8). Following removal of the heart, the free wall of the left ventricle, including the infarcted and peri-infarcted regions, was embedded in tissue-frozen medium (Thermo Fisher Scientific, Waltham, MA, USA). Frozen tissues were sectioned onto 8-μm slides and stained with hematoxylin and eosin (HE) (14). Survival of engrafted cells was confirmed by identification of DAPI-positive spots under fluorescent microscopy. Potential transdifferentiation of myocardial-like cells from implanted MSCs was verified by antibody immunostaining for rat cTnI and α-sarcomeric actin. Nesprin-1 protein expression was also verified by antibody immunostaining. Briefly, frozen tissue sections were fixed in acetone at 4°C for 10 min and incubated separately with goat anti-rat cTnI, rabbit anti-rat α-sarcomeric actin and rabbit anti-rat nesprin-1 (Abcam) for 60 min at room temperature. Following one wash with PBS solution, sections were incubated with secondary antibodies (PE-conjugated IgG) for cTnI, α-sarcomeric actin and nesprin-1 and examined under an epifluorescence microscope (Olympus BX61; Olympus Corp.).

### Identification of DAPI-labeled MSCs in vivo following transplantation

The DAPI-labeled MSCs displayed clear nuclear and faint cytoplasmic blue fluorescence when viewed under an epifluorescence microscope. The labeling efficiency of cultured MSCs with DAPI approached 100%. DAPI-labeled cells were identified in all specimens three weeks following transplantation (eight rats with transplanted MSCs at random). Three weeks following engraftment, numerous scattered DAPI-labeled cells were identified in the specimen.

### Western blot analysis

Following washing with PBS, BMSCs were removed from the culture dish and transferred to centrifuge tubes. The cardiomyocytes of the myocardial ischemia model rats were collected three weeks following MSC transplant (sham-operation group, DMEM group and MSC group). Following centrifugation at 700 xg for 10 min at 4°C, the pellets were lysed in hot Laemmli loading buffer [62.5 mmol/l Tris-HCl (pH6.8), 2% SDS, 10% glycerol, 0.05% β-mercaptoethanol and 0.05% bromophenol blue]. Equal amounts of protein extracts (20 μg/lane) were subjected to SDS-PAGE on a 5% stacking gel and 10% separating gel, followed by transfer of proteins onto a nitrocellulose membrane (20 min at 10 V). Following blocking in PBS containing 0.05% Triton X-100 and 5% FCS for 1 h, the blots were incubated overnight with rabbit anti-rat nesprin-1 at 4°C. Following washing, the membranes were incubated with the secondary antibody (horseradish peroxidase-conjugated goat anti-rabbit IgG) for 1 h and the bound antibodies were detected by enhanced chemiluminescence (Santa Cruz Biotechnology, Inc., Santa Cruz, CA, USA). β-actin was used as a control.

### Statistical analysis

Image programmer 5.1 software was used to analyze images. Values are expressed as the mean ± standard deviation. Statistical analyses were performed by paired Student’s t-tests when applicable. Statistical analysis was performed using SPSS 18.0 (SPSS, Inc., Chicago, IL, USA) and GraphPad Prism 5 Demo software (GraphPad Software, Inc., La Jolla, CA, USA).

## Results

### Characterization of MSCs and differentiated cardiomyomyte-like cells in vitro

**Figure 1:**
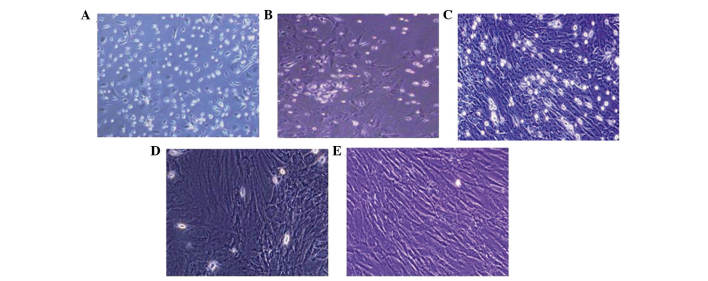
Characterization of MSCs and differentiated cardiomyocyte-like cells *in vitro*. (A) Morphological observation of rat MSCs following 3 d of culture. The cells were spindle shaped with one nucleus (magnification, ×100). (B) Morphological observation of rat MSCs following 7 d of culture (magnification, ×100). (C) Morphological observation of rat MSCs following culture for 10 d (magnification, ×100). The cells displayed as long spindle-shaped fibroblastic cells and began to form colonies (D) Morphological observation of rat MSCs following treatment with 5-azacytidine for 7 d (magnification, ×200). The cells were enlarged and assumed ball- or stick-like morphologies. (E) Morphological observation of rat MSCs following treatment with 5-azacytidine for 14 days (magnification, ×200). The cells were connected with adjoining cells. MSCs, mesenchymal stem cells.

Following discarding the non-adherent cells by the first medium change and by washing with PBS three times at 24 h of primary culture, ~80% MSCs had attached to culture dishes. The medium was subsequently changed to remove the suspended hematopoietic stem cells. Following three days of primary culture, MSCs adhered to the plastic surface and presented a small population of single cells. The cells were spindle shaped with one nucleus (Fig. 1A). Seven to ten days following initial plating, the cells developed into long spindle-shaped fibroblastic cells and began to form colonies (Fig. 1B and C). Following replating, 100% of the cells had attached to the culture dishes and were polygonal or spindle-shaped, with long processes.

**Figure 2:**
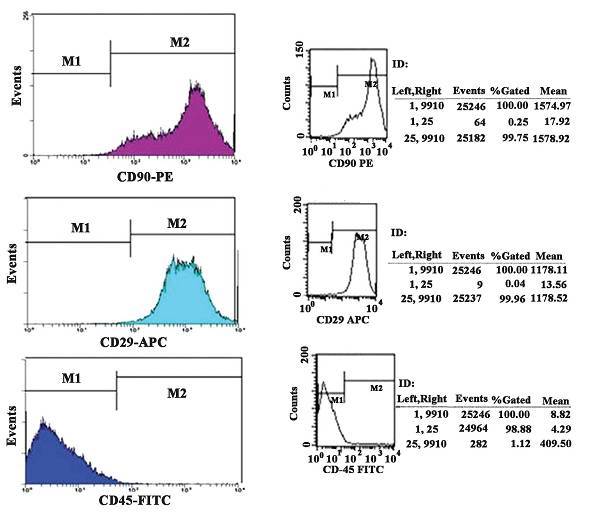
Surface-associated antigens (CD29, CD90, CD45) of MSCs were detected by flow cytometry. Surface-associated antigens of MSCs were positive for CD90, CD29 and negative for CD45. The percentage of CD90 and CD29 was ~99%, whereas the percentage of CD45 was only ~1%. MSCs, mesenchymal stem cells; PE, phycoerythrin; APC, allophycocyanin; FITC, fluorescein isothiocyanate.

Rat MSC surface antigen profiles obtained by flow cytometry (Fig. 2) were positive for CD90 and CD29 and negative for CD45. The percentages of CD90- and CD29-positive cells were 99.96 and 99.75%, respectively, whereas the percentage of CD45-positive cells was 1.12%.

The morphological differentiation from MSCs to cardiomyomytes-like cells developed gradually following 5-azacytidine induction. During exposure to 5-azacytidine, certain adherent cells died and the surviving cells began to proliferate and differentiate. One week later, ~30% of the remaining adherent cells had enlarged and assumed ball-like or stick-like morphologies. Two weeks later, the cells were observed to be connected with adjoining cells (Fig. 1D and E).

### Identification of myocardial infarction and engrafted MSCs

**Figure 3:**
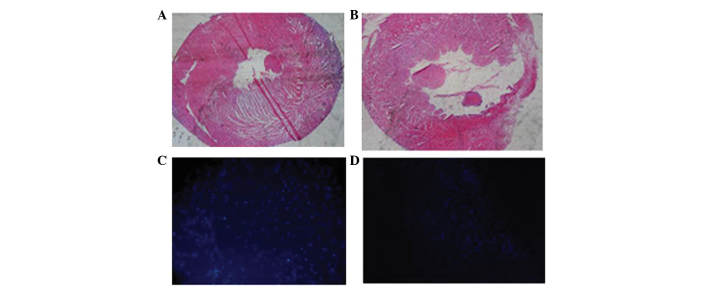
Identification of myocardial infarction and engrafted MSCs. (A) HE staining indicated the free wall of the normal left ventricle wall (magnification, ×4). (B) HE staining indicated the free wall of the left ventricle wall of myocardial infarction, which was significantly thinner than that of the normal ventricle (magnification, ×4). (C) Fluorescence image of DAPI-labeled MSCs 2 h prior to transplantation (magnification, ×40). (D) Fluorescence image of DAPI-labeled MSCs three weeks following transplantation. Dyeing efficiency of DAPI was stronger prior to transplantation (magnification, ×40). MSCs, mesenchymal stem cells; HE, hematoxylin and eosin.

HE staining of cardiac tissue obtained three weeks following myocardial infarction revealed fibrosis of the infarct region in comparison to normal cardiac tissue (Fig. 3A and B). A greater number of DAPI-positive cells were detected among the MSCs prior to transplantation (Fig. 3C) than that in the transplantation groups three weeks following transplantation (Fig. 3D), which may be due to fluorescence decay. Fig. 3D indicates engrafted cells in the ischemic myocardium, which demonstrated that implanted cells were able to survive in the peri-infarct region for three weeks post-transplantation.

### 5-azacytidine induces expression of cardiac structural proteins and mRNA in MSCs

**Figure 4:**
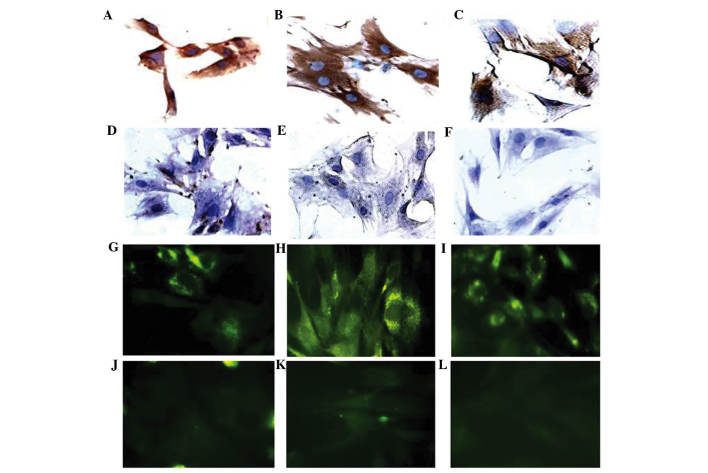
Expression of cardiac structural proteins in MSCs following 5-azacytidine treatment *in vitro*, indicated by immunohistochemical staining and immunofluorescence. (A and G) Four weeks following treatment with 5-azacytidine, MSCs were positive for desmin protein. (D and J) Untreated MSCs were negative for desmin protein following the same period. (B and H) Four weeks following treatment with 5-azacytidine, MSCs were positive for cTnI protein. (E and K) Untreated MSCs were negative for cTnI protein. (C and I) Four weeks following treatment with 5-azacytidine MSCs were positive for α-sarcomeric actin protein. (F and L) Untreated MSCs were negative for α-sarcomeric actin protein. Magnification, ×400. MSCs, mesenchymal stem cells; cTnI, cardiac troponin I.

**Figure 5:**
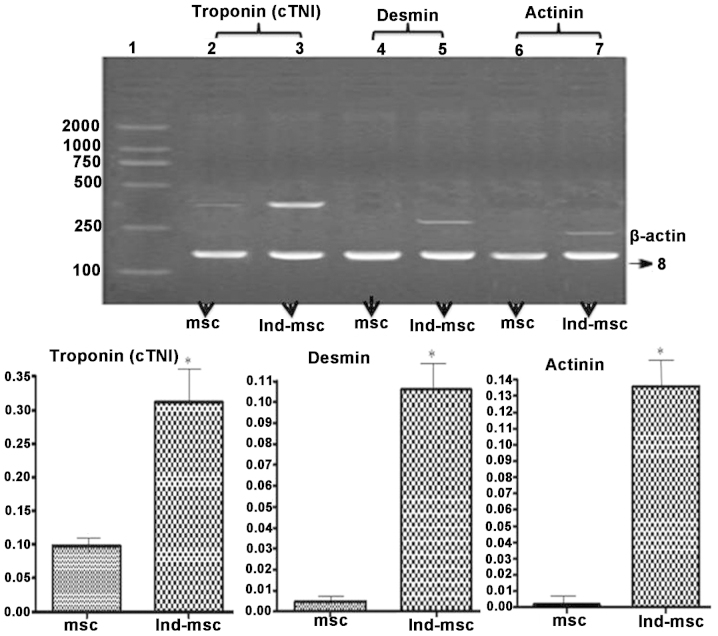
Effects of 5-azacytidine on the expression of cardiac structural protein gene expression in MSCs. Lanes: 1, marker; 2, 4 and 6, cTnI, desmin and actinin in untreated MSCs, respectively; 3, 5 and 7, cTnI, desmin and actinin of MSCs treated with 5-azacytidine, respectively. The number 8 indicates the β-actin band. MSCs induced by 5-azacytidine were positive for the expression of cTnI, desmin and actinin genes. The expression levels of cardiac structural protein genes were significantly higher in 5-azacytidine induced MSCs than those in untreated MSCs. P=0.018 (cTnI), P=0.009 (desmin), P=0.007 (actinin) vs. untreated MSCs; ^*^P<0.05 vs. the untreated MSC group. MSCs, mesenchymal stem cells; cTnI, cardiac troponin I; Ind-msc, 5-azacytidine-treated MSCs.

Immunohistochemistry and immunofluorescence assays for cTnI, desmin and α-sarcomeric actin were performed four weeks following MSC exposure to 5-azacytidine *in vitro* (Fig. 4). Untreated controls were also analyzed to confirm that there were no changes in the expression of markers of myogenic or cardiac differentiation, including the three structural proteins. Treatment of MSCs for four weeks with 10 μmol/l 5-azacytidine induced differentiation into cardiomyocyte-like cells as indicated by the expression of cTnI, actinin and desmin genes (Fig. 5). In the untreated control cells no expression of desmin, cTnI or the cardiac isoform of actinin encoded by *ACTN-2* was detected (Fig. 5).

### MSC transplantation increases the expression levels of cTnI and α-sarcomeric actin proteins in an ischemic environment

**Figure 6:**
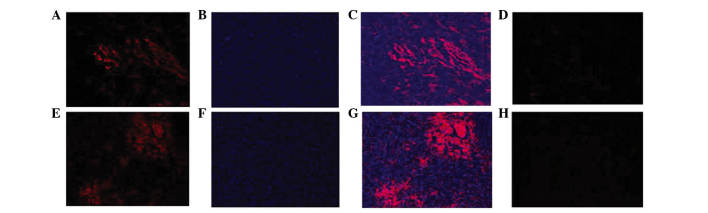
Expression of cardiac structural proteins by MSCs *in vivo,* determined by immunofluorescence. (A and E) Positive staining for cTnI and α-sarcomeric actin protein of MSCs three weeks following transplantation into an ischemic environment (red fluorescence; magnification, ×40). (B and F) DAPI-labeled nuclei of MSCs three weeks following transplantation (blue fluorescence; magnification, ×40). (C) Merged image of A and B. (G) Merged image of E and F (magnification, ×40). (D and H) Negative staining for cTnI and α-sarcomeric actin protein in myocardial infarction of Dulbecco’s modified Eagle’s medium group. MSCs, mesenchymal stem cells; cTnI, cardiac troponin I.

The expression of cTnI and α-sarcomeric actin proteins were examined in the myocardial infarction zone *in vivo,* three weeks following MSC transplant. The expression levels of cTnI and α-sarcomeric actin proteins were markedly higher in the MSC group compared with those of the DMEM group (Fig. 6).

### 5-azacytidine increases nesprin-1 expression levels in MSCs in vitro and MSC transplantation increases nesprin-1 expression levels in vivo

**Figure 7:**
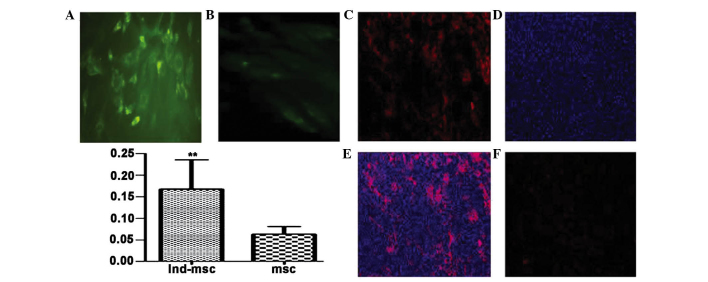
Immunofluorescence to identify the expression of nesprin-1 protein. (A) MSCs positive for nesprin-1 protein four weeks following treatment with 5-azacytidine and (B) nesprin-1 protein expression of untreated MSCs following four weeks of culture (green fluorescence; magnification, ×400; ^**^P=0.0032 vs. untreated group). (C) MSCs positive for nesprin-1 protein three weeks following transplant into an ischemic environment (red fluorescence; magnification, ×40). (D) DAPI-labeled nuclei of transplanted MSCs three weeks following transplantation (blue fluorescence; magnification, ×40). (E) Merged image of C and D (magnification, ×40). (F) Negative staining for nesprin-1 protein in myocardial infarction of Dulbecco’s modified Eagle’s medium group. MSCs, mesenchymal stem cells; Ind-msc, 5-azacytidine-treated MSCs.

Immunofluorescent staining for nesprin-1 protein expression verified the presence of the transplanted rat MSCs (Fig. 7A and B). Nesprin-1 protein expression levels were significantly higher in the MSCs treated with 10 μmol/l 5-azacytidine *in vitro* for four weeks than those in the untreated MSCs.

The results displayed in Fig. 7C and E indicated that nesprin-1 protein expression levels were markedly higher in the MSC group in comparison with those in the control group. The expression of nesprin-1 protein in the myocardial infarction zone was detected by immunofluorescence three weeks following MSC transplantation.

### Nesprin-1 protein expression indicates MSC differentiation

**Figure 8:**
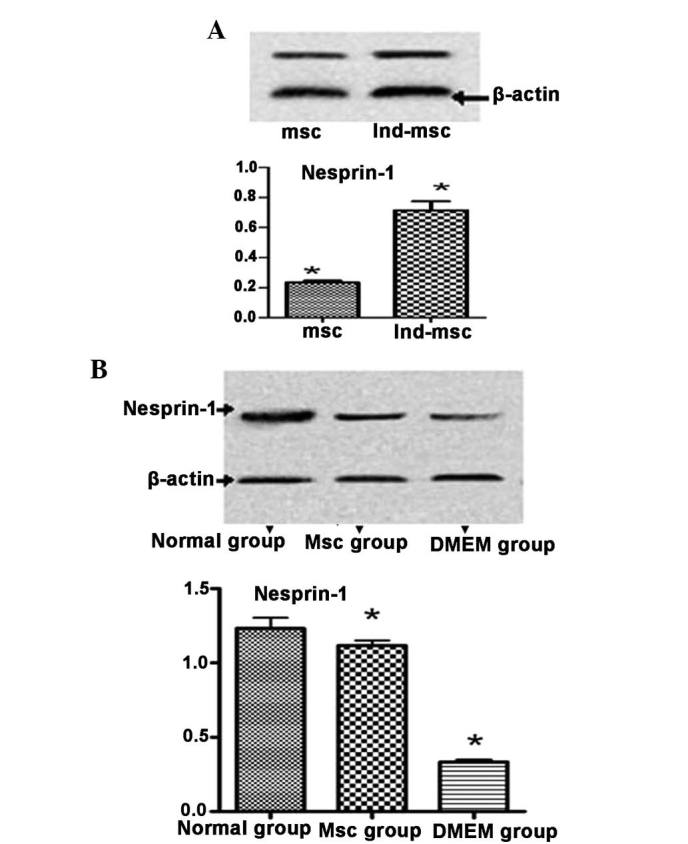
Expression levels of nesprin-1 protein detected by western blot analysis. (A) Expression levels of nesprin-1 protein in the 5-azacytidine-treated MSCs are higher than those of the untreated MSCs. (B) Nesprin-1 protein expression levels were higher in the MSC group than those in the DMEM control group, but lower than those in the normal group. MSCs, mesenchymal stem cells; DMEM, Dulbecco’s modified Eagle’s medium; ind-msc, 5-azacytidine-treated MSCs; Ind-msc, 5-azacytidine-treated MSCs.

Nesprin-1 protein expression levels were higher in the MSC group than those in the DMEM control group, but lower than those in the normal group (Fig. 8B). Treatment of MSCs for four weeks with 10 μmol/l 5-azacytidine induced their differentiation into cardiomyocyte-like cells, confirmed by the significantly higher expression levels of nesprin-1 protein in the 5-azacytidine-treated MSCs compared with those in the untreated MSCs.

## Discussion

MSCs were first described in 1968 by Friedenstein *et al* (15). These cells can be expanded *ex vivo* and induced, either *in vitro* or *in vivo*, to terminally differentiate into osteoblasts, chondrocytes, adipocytes, tenocytes, myotubes, neural cells and hematopoietic cells with strong self-renewal ability and genetic stability *in vitro* (6). Several studies reported that MSCs were able to proliferate and potentially differentiate *in vitro* (16–18). However, the ratio of MSCs in bone-marrow is only ~0.001–0.01%. Hence, the separation and amplification of MSCs is of vital importance. Wakitani *et al* (19) described a method to isolate MSCs from rat bone marrow using Ficoll density gradient separation and adherent culture. The International Society for Cellular Therapy proposed three minimal criteria to identify MSCs: *i*) MSCs must be plastic-adherent if maintained in standard culture conditions; *ii*) MSCs must express CD105, CD73 and CD90, but lack haematopoietic markers, including CD45, CD34, CD14 or CD11b, and *iii*) MSCs must be capable of differentiating into fibroblasts, osteoblasts, adipocytes and chondroblasts under the corresponding lineage, particularly under *in vitro* conditions (20). The MSC surface antigens CD90 and CD29 were detected by flow cytometry; the percentage of CD90 and CD29 detected was ~98%, whereas the percentage of CD45 was ~1%.

Xu *et al* (21) reported that the ability of human MSCs to proliferate remained strong between passages two and six. Therefore, second-passage rat MSCs were selected for the present study, to investigate whether these cells were able to differentiate into cardiomyocyte-like cells *in vitro* following 5-azacytidine treatment. Makino *et al* (22) and Toma *et al* (23) reported that following 5-azacytidine treatment, rat MSCs differentiated into cardiomyocyte lineages *in vivo* and *in vitro*. These MSCs developed into cardiomyocyte-like cells, which expressed the cardiac myocyte markers, myosin heavy chain and troponin T in cardiomyocyte medium subjected to hypoxia re-oxygenation and treatment with hepatocyte growth factor, bone morphogenetic protein 2 and fibroblast growth factor 4 (24–26). Li *et al* (27) reported a localization of cardiac troponin T (cTnT) in DAPI-labeled B-cell lymphoma-2-transduced MSCs in a rat model of irreversible ligation of the left anterior descending coronary artery, indicating differentiation towards cardiomyocyte-like cells. In the present study, 10 μmol/l 5-azacytidine was used to induce rat MSCs to differentiate into cardiomyocyte-like cells *in vitro*, which led to the adherent cells enlarging and assuming ball-or stick-like morphologies. Four weeks later, the expression of cTnI, actinin and desmin genes was detected in cardiomyocyte-like cells by RT-qPCR. Expression of the proteins cTnI, α-sarcomeric actin and desmin was also identified by immunofluorescent staining.

Hu *et al* (28) reported that implanted MSCs were able to survive in the peri-infarct region for ≥four weeks post-transplantation when the MSCs were traced using DAPI. The greatest number of DAPI-positive cells was detected in the myocardium and the greatest functional benefit was observed when MSCs were transplanted one week following myocardial infarction. In the present study, DAPI was applied to label and trace MSCs which were engrafted two weeks following myocardial infarction. Three weeks following MSC transplant into the site of myocardial infarction, expression of cTnI and α-sarcomeric actin was detected.

Although positive results have been obtained in cell-based therapies to treat myocardial infarction, the underlying mechanisms have remained to be elucidated. Therefore, in the present study the expression of nesprin-1 protein prior to and following MSC differentiation was also examined. The results revealed that nesprin-1 protein expression was higher in cardiomyocyte-like cells than that in undifferentiated MSCs.

Nesprins are a family of proteins that bind to the nuclear envelope (NE) and interact with emerin and lamin A/C (2,4,29,30). The structure of nesprin isoforms suggests that they form a protein scaffold linking the NE to the nucleus, cytoplasmic organelles and cell membrane via the actin cytoskeleton (29,30). These studies suggested a role for nesprin in the structural organization of the sarcomere and signaling between the extracellular environment and nucleus (31,32). Gough *et al* (33) concluded that the Syne-1 (nesprin-1) gene was expressed in a variety of forms that are multifunctional and capable of functioning at the Golgi and the NE, including linkage of the two organelles during muscle differentiation.

High expression levels of nesprin-1 were observed in skeletal, cardiac and vascular smooth muscle. Zhang *et al* (2) suggested that nesprin-1 may have specific functions in muscle cell differentiation; however, high expression levels of nesprin-1 were detected in peripheral blood leukocytes and the spleen. Nesprin-1 is highly expressed in muscle tissue and has muscle-specific isoforms (2,4,30). During *in vitro* differentiation of C2C12 myoblasts into myotubes, nesprin-1 localization shifts from the nuclei/NE to the cytoplasm/sarcomere, indicating a specific role in muscle differentiation (2,4). In the sarcomere of skeletal muscle cells, various nesprin-1 epitopes are associated with the Z-line, A/I junction, sarcoplasmic reticulum and mitochondrial membrane, indicating that nesprin-1 may contribute to sarcomeric structure maintenance (4,34). Furthermore, sarcomeric proteins have been identified as potential interaction partners for nesprin-1 and −2, including the ryanodine receptor and muscle-specific A-kinase anchoring protein (mAKAP). mAKAP is targeted to the NE by nesprin-1 and they interact through their closely associated sarcoplasmic reticulums. Nesprins may potentially be involved in maintaining and targeting protein complexes common to the NE and the sarcoplasmic reticulum (4,35). A study demonstrated that cardiomyocyte nuclei were elongated with reduced heterochromatin in Delta/DeltaKASH mouse hearts (36). These findings reflected the results of previous studies on lamin A/C gene mutations and therefore reinforced the importance of an intact nuclear membrane complex for a regularly functioning heart (36). During the development of immature to mature muscle fibers *in vivo*, nesprin-2 was partially replaced by nesprin-1 at the NE and short nesprin isoforms became dominant. In emerin-negative skin fibroblasts, nesprin-2-giant was relocated from the NE to the cytoplasm, while nesprin-1 remained at the NE (37).

Nesprin-1 may therefore have a key function in addition to its characterized roles in cell mitosis, RNA copy of transport and the stability of the nuclear membrane. Nesprin-1 may also have a significant role in cell differentiation. In the present study, it was revealed that the expression levels of nesprin-1 protein were higher in the infarcted myocardium implanted with MSCs than those of the control group. In conclusion, it was hypothesized that nesprin-1 had an important role in mediating the differentiation of MSCs into cardiomyocyte-like cells. These results may provide an experimental base from which to improve cell-based therapies for the treatment of myocardial ischemia.

## Figures and Tables

**Table I:** Primers used for reverse transcription quantitative polymerase chain reaction.

Name	Primer sequence	Length (bp)
α-actinin	F: 5′-TGGTCTTGGTTTCTGTGCCTTG-3′ R: 5′-CTGCTGTTTCCGCCTTCTGG-3′	251
Desmin	F: 5′-AATGACCGCTTCGCCAACTAC-3′ R: 5′-TATCAGGTTGTCACGCTCCACG-3′	207
Cardiac troponin I	F: 5′-AAGCAGGAGATGGAGCGTGAG-3′ R: 5′-TCCTCCTTCTTCACCTGCTTG-3′	368

F, forward; R, reverse.
